# Bacterial and Fungal Toll-Like Receptor Activation Elicits Type I IFN Responses in Mast Cells

**DOI:** 10.3389/fimmu.2020.607048

**Published:** 2021-02-12

**Authors:** Lisa Kornstädt, Sandra Pierre, Andreas Weigert, Stefanie Ebersberger, Tim J. Schäufele, Anja Kolbinger, Tobias Schmid, Jennifer Cohnen, Dominique Thomas, Nerea Ferreirós, Bernhard Brüne, Ingo Ebersberger, Klaus Scholich

**Affiliations:** ^1^Institute of Clinical Pharmacology, University Hospital Goethe-University Frankfurt, Frankfurt, Germany; ^2^Faculty of Medicine, Institute of Biochemistry I, Goethe-University Frankfurt, Frankfurt, Germany; ^3^Institute of Molecular Biology gGmbH, Mainz, Germany; ^4^Project Group Translational Medicine and Pharmacology, Fraunhofer Institute for Molecular Biology and Applied Ecology IME, Frankfurt am Main, Germany; ^5^Department for Applied Bioinformatics, Institute for Cell Biology and Neuroscience, Goethe-University Frankfurt, Frankfurt, Germany; ^6^Senckenberg Biodiversity and Climate Research Centre (S-BIKF), Frankfurt am Main, Germany; ^7^LOEWE Centre for Translational Biodiversity Genomics (TBG), Frankfurt am Main, Germany; ^8^Fraunhofer Cluster of Excellence for Immune-Mediated Diseases (CIMD), Frankfurt am Main, Germany

**Keywords:** mast cells, inflammation, resolution, IFN-β, toll-like receptor, phagocytosis

## Abstract

Next to their role in IgE-mediated allergic diseases and in promoting inflammation, mast cells also have antiinflammatory functions. They release pro- as well as antiinflammatory mediators, depending on the biological setting. Here we aimed to better understand the role of mast cells during the resolution phase of a local inflammation induced with the Toll-like receptor (TLR)-2 agonist zymosan. Multiple sequential immunohistology combined with a statistical neighborhood analysis showed that mast cells are located in a predominantly antiinflammatory microenvironment during resolution of inflammation and that mast cell-deficiency causes decreased efferocytosis in the resolution phase. Accordingly, FACS analysis showed decreased phagocytosis of zymosan and neutrophils by macrophages in mast cell-deficient mice. mRNA sequencing using zymosan-induced bone marrow-derived mast cells (BMMC) revealed a strong type I interferon (IFN) response, which is known to enhance phagocytosis by macrophages. Both, zymosan and lipopolysaccharides (LPS) induced IFN-β synthesis in BMMCs in similar amounts as in bone marrow derived macrophages. IFN-β was expressed by mast cells in paws from naïve mice and during zymosan-induced inflammation. As described for macrophages the release of type I IFNs from mast cells depended on TLR internalization and endosome acidification. In conclusion, mast cells are able to produce several mediators including IFN-β, which are alone or in combination with each other able to regulate the phagocytotic activity of macrophages during resolution of inflammation.

## Introduction

Mast cells are part of the first line of defense of the body, protecting against invading pathogens and other environmental harm. They are long-lived, tissue-resident leukocytes, located most abundantly close to surfaces exposed to the environment, like skin and mucosal tissues. Their most characteristic feature is the secretory granules densely packed with pre-formed mediators, which can be released rapidly upon activation by degranulation. Mast cell activation can also induce *de novo* synthesis and release of lipid mediators (e.g. prostaglandin (PG) E2 and thromboxane), cytokines and chemokines (1, 2). The precise response and signaling pathway activated in a mast cell depends on the stimulus activating the cell, which can be recognized and discriminated by an extensive repertoire of receptors. These receptors coordinate the selective release of proinflammatory (e.g. histamine, interleukin (IL)-1β) or antiinflammatory (e.g. IL-4, IL-10, IL-13) mediators (1, 3).

To detect invading pathogens, mast cells express a variety of pattern recognition receptors (PRRs), including Toll-like receptors (TLRs). TLRs are transmembrane proteins located at the cell surface or in intracellular compartments like endosomes or lysosomes. They form homo- or heterodimers and recruit a set of adaptor molecules such as MyD88 and TRIF for signaling (4, 5). While MyD88 is known to be utilized by all TLRs except for TLR3, TRIF is considered to be selectively recruited to TLR3 and TLR4. The MyD88-dependent pathway leads to the activation of the NF-κB pathway and the MAPK pathway, resulting in the induction of pro-inflammatory cytokines like IL-1β. The TRIF-dependent pathway depends on TLRs present on endosomes and mediates the induction of type I IFNs and IFN-inducible genes by activating transcription factors of the IFN regulatory factor (IRF) family (4, 5). Notably, intracellular localization of TLR4 can also be required for the MyD88-dependent pathway (6).

As part of the anti-viral response, e.g. against SARS-CoV-2, mast cells release a specific set of proinflammatory mediators, including IL-1 and IL-6, which are positively associated with COVID-19 severity (7, 8). Also type I IFNs were originally described to be induced as an anti-viral response but are also produced in response to bacterial pathogens (9). The family of type I IFNs consists of at least 13 IFN-α isotypes, one IFN-β isotype and various others. The cytosolic TLR receptors TLR3, TLR7, and TLR9 sense viral nucleic acids and respond by initiating the type I IFN response. TLR4 activates the MyD88-dependent NF-κB pathway while located in the plasma membrane and starts type I IFN signaling through TRIF when internalized into endosomal compartments. Induction of the type I IFN response by TLR2 also takes place from endosomal compartments, but in a MyD88-dependent pathway (10–12).

Here we aimed to investigate the potential roles of antiinflammatory mediators released by mast cells during the resolution phase of a local zymosan-induced inflammation. Zymosan-induced inflammation is a widely used model for local short-lasting inflammation and evokes well-defined inflammatory and behavioral responses. In an untargeted mRNA sequencing approach to identify transcriptional changes in mast cells in response to the TLR2 ligand zymosan, we found strong upregulation of genes involved in the type I IFN response. We further showed that mast cells produce type I IFNs also in response to LPS, which activates TLR4. This was surprising since mast cells were believed to lack the ability to produce type I IFNs as answer to TLR4 activation, due to being unable to internalize surface-bound pathogens (13, 14). Furthermore, as previously shown for macrophages (12, 13) the induction of the type I IFN response is elicited from endosomal compartments also in mast cells.

## Materials and Methods

### Animals

C57BL/6N mice were supplied by Janvier (Le Genest, France). Mcpt5-DTA-Cre mice were originally described and provided by Professor Axel Roers, Technische Universität Dresden, Germany (15). Sex and age matched Mcpt5-DTA-Cre^-^ litter mates were used as control mice for Mcpt5-DTA-Cre^+^ mice. The animals were cared for according to the International Association for the Study of Pain guidelines (Grants FK1066, FK1093, and FK1138). For all experiments, the ethics guidelines for investigations in conscious animals were obeyed and the procedures were approved by the local ethics committee (Regierungspräsidium Darmstadt). The animals had free access to food (Sniff standard diet) and water and were maintained in climate- (23°C ± 0.5°C) and light-controlled rooms (light from 6.00 a.m. to 6.00 p.m.).

### Behavioral Tests

Inflammation was induced by injection of 10 µl zymosan (12 mg/ml in PBS, #Z4250, Sigma-Aldrich) subcutaneously into the plantar side of one hind paw. Mechanical hypersensitivity was determined by measuring the latency of paw withdrawal using a plantar aesthesiometer (Dynamic Plantar Aesthesiometer, Ugo Basile). A force range of 0 to 5 g with a ramp of 0.5 g/s was applied with a steel rod of 2 mm in diameter, until a strong and immediate withdrawal occurred. The cutoff time was set to 20 s.

### Multi Epitope Ligand Cartography (MELC)

MELC technology is an automated immunohistological imaging method and can be used to visualize very high numbers of antibodies on the same sample as described before (16–18). Briefly, tissues were embedded in tissue freezing medium (Tissue-Tek O.C.T. Compound, #4583, Sakura Finetek B.V.), cryosections of 10 µm thickness were applied on silane-coated coverslips, fixed in 4% paraformaldehyde in PBS for 15 min, permeabilized with 0.1% Triton X100 in PBS for 15 min and blocked with 3% BSA in PBS for 1 h. The sample was placed on the stage of a Leica DM IRE2 and a picture was taken. Then, in an automated procedure, the sample was incubated for 15 min with bleachable fluorescence-labeled antibodies and rinsed with PBS. Afterward, the phase contrast and fluorescence signals were imaged by a cooled charge-coupled device camera (Apogee KX4, Apogee Instruments). A bleaching step was performed to delete fluorescence signals, and the post-bleaching image was recorded. Then the next antibody was applied and the process repeated. For data analysis, fluorescence images produced by each antibody were aligned pixel-wise and were corrected for illumination faults using flat-field correction. The post-bleaching images were subtracted from their following fluorescence image. The antibodies used for MELC analysis were against CD11b (BioRad, #MCA74F), CD11c (Miltenyi Biotec, #130-102-799), CD31 (BD Biosciences, #553373), CD45 (Miltenyi Biotec, #130-091-609), CD54 (Biolegend, #116105), CD80 (Biolegend, #104706), CD86 (Biolegend, #105008), CD117 (Bioss, #bs-10005R-Cy5), CD206 (AbDSerotec, #MCA22335FA), F4 80 (Biolegend, #123107), IL-1β (Thermo Fisher, #11-7114-82), IL-4 (Biolegend, #504109), IL-10 (eBioscience, #11-7101), IL-13 (Invitrogen, #53-7133-82), Ly6C (eBioscience, #17-5932-80), Ly6G (eBioscience, #11-5931-82), MHC II (Miltenyi Biotec, #120-000-810), Siglec F (BD Pharmingen, #562068). Nuclei were stained with propidium iodide (PI) (Sigma-Aldrich, #P4170)

### Analysis of MELC Data

In a first step all greyscale antibody channel images were processed using ImageJ 1.52v to diminish noise, background fluorescence and remove artifacts for further analyses if necessary. Subsequently, images for propidium iodide (cell nuclei) and CD45 were used for single-cell segmentation using Cell Profiler (version 3.1.9) (19). The resulting segmentation mask was loaded into histoCAT (version 1.76) (20) together with the corresponding antibody channel images. All images, excluding zymosan images and images used for single-cell mask generation, were z-score normalized and used for Barnes-Hut t-SNE (BH t-SNE) (21) and PhenoGraph analysis (22) as implemented in histoCAT. PhenoGraph defines cell clusters based on single-cell mask and marker colocalization (k was set to 20 or 30). BH t-SNE scatter plot was overlaid with a colored PhenoGraph cluster map. To investigate the relationship between clusters, neighborhood analysis under standard conditions as implemented in histoCAT was used (20).

### Multiplex Cytokine Assay

Cytokine and chemokine levels were determined in ipsi- and contralateral paws of Cre^-^ and Cre^+^ Mcpt5-DTA mice 48 h after injection of 10 µl 12 mg/ml zymosan, using the Bio-Plex Pro mouse cytokine group I (Bio-Rad). Tissue samples of paws were dissected and frozen directly at −80°C until protein extraction. The tissue was lysed in 400 µl lysis buffer (1x Protease Inhibitor Cocktail (#11697498001, Roche) in Tissue Extraction Reagent (#FNN0071, Invitrogen). Samples were cut in small pieces and then sonicated twice at 60% power for 10 sec with an Ultrasonic Homogenizer (SONOPULS HD2070 MS73, Bandelin). Afterwards all samples were centrifuged for 10 min at 10,000 g and the supernatant harvested. The concentration of total protein in the samples was assessed by the bicinchoninic acid assay. All samples were diluted to a final protein concentration of 200–900 µg/ml, according to the kit requirements. The concentrations of IL-1α, IL-1β, IL-2, IL-3, IL-4, IL-5, IL-6, IL-9, IL-10, IL-12p40, IL12-p70, IL-13, IL-17, Eotaxin, G-CSF, GM-CSF, IFN-*γ*, CXCL1 (KC), CCL2 (MCP-1), CCL3 (MIP-1α), CCL4 (MIP-1β), CCL5 (RANTES), TNF-α were measured with a Bioplex 200 (Bio-Rad). The concentration was then normalized to the total protein concentration of the respective sample and is shown as pg/mg protein.

### Polychromatic Flow Cytometry

Mcpt5-DTA Cre^-^ and Cre^+^ mice were injected with 10 µl zymosan (12 mg/ml #Z4250, Sigma-Aldrich) and pHrodo Red zymosan Bioparticles (#P35364, Invitrogen) per paw into the plantar side of one hind paw per mouse. Polychromatic flow cytometry was performed essentially as described previously (23). Briefly, single-cell suspensions were generated from solid tissues (<1 mm^3^), by digestion with 3 mg/ml Collagenase IA (Sigma) DMEM for 45 min at 37°C, followed by filtration through a 70 µm nylon mesh (BD Life Sciences). Then the cells were incubated for 5 min in DMEM containing 10% FCS to stop the lysis followed by incubation in ACK buffer for 5 min. After centrifugation (1,000 g, 5 min), the cells were washed in PBS and resuspended in 30 µl FACS buffer (1% FCS in PBS), followed by incubation with an antibody cocktail for 1 h at 4°C. Cells were transferred to FACS tubes. Samples were acquired with a FACS Canto II flow cytometer and analyzed using FlowJo software V10 (both BD Biosciences). For gating, fluorescence minus 1 (FMO) controls were used. The antibodies used for FACS analysis were: CD117-PE (Miltenyi, #130-102-795), FcϵRIα-FITC (#134305, Biolegend), Ly6C-eFluor450 (eBioscience #48-5932-82) Ly6G/Ly6C-FITC (eBioscience, #11-5931-82), F4/80-PE/Cy7 (Biolegend, #123113), F4/80-PE (Biolegend, #123110), SiglecF-PE-Vio770 (Miltenyi, #130-102-167), CD45-VioGreen (Miltenyi, #130-123-900), CD206-APC (Biolegend, #141708), CD86-BrilliantViolet421 (Biolegend, #105031), CD11b-APC/Cy7 (Biolegend, #101225), MHCII-FITC (Miltenyi, 130-081-601). For analysis of phagocytosed neutrophils, intracellular staining with Ly6G-APC (Biolegend, #127614) was performed. Cells were prepared as described above, incubated with ACK buffer, fixed for 5 min with 1% PFA and washed with PBS. Then the cells were permeabilized with 0.1% Saponin and incubated for 1 h at 4°C with antibodies in FACS buffer containing 0.1% Saponin. After the incubation, the cells were washed with 0.1% Saponin and resuspended in PBS for flow cytometry analysis.

### Bone Marrow-Derived Mast Cells (BMMCs)

BMMCs were prepared as described earlier (24). Bone marrow cells were isolated from murine femur and tibia from the hind legs of adult mice. The bones were cut open at one end and centrifuged at 10,000 g for 1 min. The cells of a single animal were resuspended in 40 ml of mast cell medium consisting of RPMI 1640 medium supplemented with 10% fetal bovine serum, 100 U/ml penicillin/streptomycin, 4 mM L-Glutamine, 1 mM sodium pyruvate, 1% MEM nonessential amino acids, 50 µM 2-mercaptoethanol and 10 µg/L IL-3 (#213-13, PeproTech). Forty milliliters of medium was added twice a week. The cells were cultivated for 4 to 6 weeks at 37°C with 5% CO_2_ under humidified conditions. After 4–6 weeks, the purity and maturity of the mast cells was assessed by FACS analysis on a FACS Canto II flow cytometer (BD Life Sciences). For FACS analysis, a portion of the cells was centrifuged at 500 g for 10 min, washed with PBS and then resuspended in FACS buffer (1% FCS in PBS). The cells were then incubated for 1 h with CD117-PE (130-102-795, Miltenyi Biotec) and FcϵRIα-FITC (#134305, Biolegend) at 4°C, washed and analyzed on a FACS Canto II flow cytometer (BD Life Sciences) (**Figure S1**).

### Bone Marrow-Derived Macrophages (BMDMs)

BMDMs were prepared as described earlier (16). Bone marrow cells were isolated from murine femur and tibia from the hind legs of adult mice. The bones were cut open at one end and centrifuged at 10,000 g for 1 min. The cells were differentiated in macrophage medium consisting of RPMI 1640 GlutaMAX medium supplemented with 10% fetal bovine serum, 100 U/ml penicillin/streptomycin, and 20 ng/ml macrophage colony stimulating factor (M-CSF, #AF-315-02, Peprotech). Non-adherent cells were removed after one day of cultivation by exchange of medium, fresh medium was added after 4 days. Cells were grown for 7 days at 37°C and 5% CO2. Non-adherent cells were removed after one day of cultivation by exchange of medium, fresh medium was added after 4 days.

### RNA Sequencing

BMMCs were cultivated for 4–6 weeks as described, then the cell number was adjusted to 8x10^5^ cells/ml and the cells were treated with 10 µg/ml zymosan for 24 h or 48 h or left untreated (0 h). Cells were stained as described above and then sorted using a FACS Aria (BD Biosciences) to obtain a pure population of CD117^+^/FcϵRIα^+^ double-positive cells. RNA was isolated from the FACS-sorted cells using the RNeasy micro Kit (Qiagen, #74004). Library preparation was carried out with the QuantSeq 3’ mRNA Library Prep Kit FWD (Lexogen). The quality of the libraries was controlled with a Bioanalyzer High Sensitivity DNA Assay (Agilent Technologies), quantification was carried out using a Qubit dsDNA HS assay (Thermo Fisher Scientific). Sequencing was performed on a NextSeq500 using a NextSeq500 High Output (75 cyc) Kit (Illumina).

The raw sequencing reads were preprocessed with the software bcl2fastq. Read mapping against the mouse reference genome (GRCm38.p6, primary assembly) was done with the STAR aligner (version 2.6.0a 2018/04/23) (25). The mapped reads were assigned to annotated features (genes) using the Bioconductor package Rsubread (v1.28.1) (26). Unassignable reads comprise reads overlapping with more than one gene (ambiguous), non-unique mappers and reads that map outside known genes. On average 75% of uniquely mapped reads could be assigned to genes. Differential expression of genes between conditions was assessed with the Bioconductor package DESeq2 (v1.18.1) (27). Gene ontology analysis was performed with the gene set library “GO Biological Processes 2018” by EnrichR (28, 29).

### Cytokine Measurements

BMMCs were cultivated for 4–6 weeks as described, then the cell number was adjusted to 8x10^5^ cells/ml in mast cell medium and the cells were pre-incubated and induced as indicated in the respective figure legends with 25 µM Cytochalasin D (#C2618, Sigma-Aldrich), 50 µM Bafilomycin A (#SML1661, Sigma-Aldrich), and 10 µg/ml zymosan (#Z4250, Sigma-Aldrich) or 100 ng/ml LPS (#L3129, Sigma-Aldrich). BMDMs were cultivated for 7 days as described and induced with 10 µg/ml zymosan. Cytokines were measured at the indicated time points after induction using the following ELISA Kits: Mouse IL-10 Quantikine (#M1000B), Mouse IL-1 beta/IL-1F2 Quantikine (#MLB00C), Mouse IFN-beta Quantikine (#MIFNB0, all from R&D Systems), VeriKine-HS (High Sensitivity) Mouse IFN Alpha All Subtype (#42115-1, PBL Assay Science).

### LC-MS/MS

LC-MS/MS analysis of PGE_2_ in BMMC culture medium was performed as described previously (24). Briefly, 100 µl PBS, 100 µl 150 mM EDTA, and 20 µl internal standard solution (10 ng/ml of [^2^H_4_]-PGE_2_ in methanol) were added to 100 µl cell suspension before extraction liquid-liquid extraction using with 600 µl ethyl acetate. Organic layer was separated and the extraction was repeated using again 600 µl ethyl acetate. The organic layers were combined, evaporated at 45°C under a gentle stream of nitrogen and reconstituted with 50 µl of acetonitrile:water:formic acid (20:80:0.0025, v/v). 10 µl of this solution were injected into the LC-MS/MS system. For LC-MS/MS analysis an Agilent 1290 Infinity LC system (Agilent, Waldbronn, Germany) coupled to a hybrid triple quadrupole linear ion trap mass spectrometer QTRAP 6500+ (Sciex, Darmstadt, Germany) equipped with a Turbo-V-source operating in negative ESI mode was used. Chromatographic separation was done using a Synergi Hydro-RP column (2.0 x 150 mm, 4 µm particle size; Phenomenex, Aschaffenburg, Germany), coupled to a precolumn of the same material. 0.0025% formic acid and acetonitrile containing 0.0025% formic acid served as mobile phases. Mass spectrometric parameters were: Ionspray-voltage −4,500 V, source temperature 500°C, curtain-gas 40 psi, nebulizer-gas 40 psi, Turboheater-gas 60 psi. Both quadrupoles were running at unit resolution. For analysis, Analyst Software 1.6 and Multiquant Software 3.0 (both Sciex, Darmstadt, Germany) were used, employing the internal standard method (isotope-dilution mass spectrometry).

### Statistical Analysis

Statistical significance was determined by unpaired t-test, one-way ANOVA using a Tukey’s post-test or Dunnett’s post-test, or two-way ANOVA using a Sidak’s post-test through the GraphPad Prism 6 software as outlined in the figure legends.

## Results

### Mast Cells Influence Macrophage Phagocytosis During Resolution of Zymosan-Induced Inflammation

**Figure 1:**
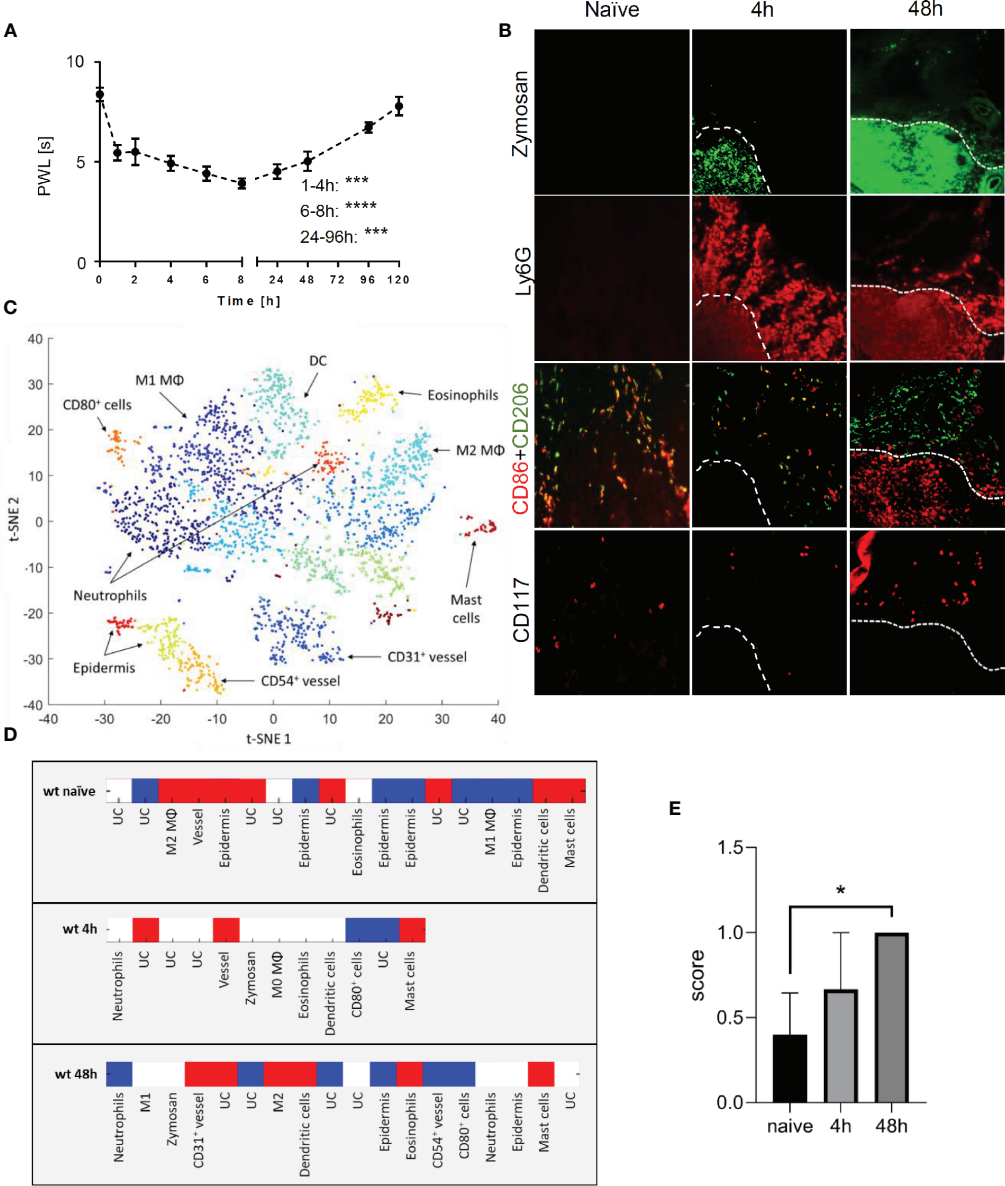
Mast cells are located in antiinflammatory regions during resolution phase of zymosan-induced inflammation. **(A)** Mechanical paw withdrawal latencies in mice (n=10) at indicated time points after injection of zymosan (10 µl, 12 mg/ml in PBS). Data are mean ± S.E.M., one-way ANOVA, Dunnett’s test vs. baseline, ***p<0.001, ****p<0.0001. **(B)** Representative images of immunohistological multi epitope ligand cartography (MELC) staining of paws form naïve mice or 4 h or 48 h after injection of zymosan. FITC-labeled zymosan, marker for pro-inflammatory cells (CD86), anti-inflammatory cells (CD206), mast cells (CD117) are shown in false colors. White dotted lines depict the outline of the zymosan-filled area in the paw. **(C)** Representative Barnes-Hut t-SNE (BH t-SNE) plot colored by cell clusters defined by PhenoGraph analysis. Underlying images originate from MELC analysis of mice paw 48 h after zymosan injection. The positions of some cell types are depicted in the plot. DC, dendritic cells; MΦ, macrophages. **(D)** Heat map showing a representative result of the neighborhood analysis for mast cells in naïve paw and 4 h or 48 h after zymosan injection (n=3–4). UC, unidentified cluster. Red depicts cells, which neighbor mast cells more frequently than they would in random permutations of cell cluster labels in each image set. Blue depicts cell clusters neighbor less frequently than with randomly permuted cell labels and white depicts cells cluster neighbors with random frequency. **(E)** Averaged score for mast cell neighborhood regarding antiinflammatory macrophages (M2 MΦ) based on neighborhood analysis of naïve paw or 4 h or 48 h after injection of zymosan (n=3-4). A score was assigned for red=1, white=0, blue=−1. Data are shown as mean ± S.E.M., one-way ANOVA test, *p<0.05.

To study the time course of the inflammatory response to the TLR2 ligand zymosan, we determined the mechanical hypersensitivity after subcutaneous injection of zymosan into the plantar side of one hind paw. Mechanical hypersensitivity increased significantly as early as 1 h after zymosan injection and was maintained for at least 8 h after injection. Afterwards hypersensitivity receded until it returned to baseline at day 5 (**Figure 1A**). The receding mechanical hypersensitivity is indicative for the resolution phase of the inflammation. To study the localization of mast cells and to determine their neighboring cells during resolution of inflammation, MELC analyses were performed with paws from naïve mice as well as 4 and 48 h after injection of zymosan into the paw. Throughout the time-course there was no significant increase of the mast cell number (**Figures S2A, B**). The MELC technology is an automated system, allowing the sequential imaging of an unrestricted number of directly labeled antibodies on the same tissue sample. To enable visualization of zymosan in the paw FITC-labeled zymosan was used for injection. At peak inflammation 4 h after zymosan injection, a time point with early inflammation and hypersensitivity, neutrophils (Ly6G^+^) were located in and around the zymosan-containing region (**Figure 1B**). In addition the appearance of CD86^+^/CD206^+^ macrophages outside of the zymosan-containing region was observed. Forty-eight hours after zymosan injection, a time point with declining mechanical hypersensitivity, neutrophils (Ly6G^+^) were located mainly in the same region as zymosan (**Figure 1B**) and proinflammatory CD86^+^ cells were located in or near the zymosan-containing area. Antiinflammatory CD206^+^ cells surrounded the region containing the CD86^+^ cells (18). Quantitative analysis of the MELC data was performed using a machine learning approach using HistoCAT software (20), to determine individual cell phenotypes and their cellular microenvironments. Single-cell segmentation was performed based on staining for CD45 and nuclei, which was followed by PhenoGraph analysis allowing the discrimination of the different immune cell types including mast cells (**Figure 1C**). PhenoGraph analysis of MELC images for the relative location of 21 antibodies showed expression of cytokines with certain antiinflammatory properties (IL-4, IL-13, and IL-33) as well as the proinflammatory cytokine IL-1β in mast cells (**Figure S3**). To determine the cellular neighborhood of mast cells, a neighborhood analysis for all identified mast cells was performed. This analysis aims to determine which cell types are neighboring mast cells more often as expected for a random distribution (20). The analysis revealed that 48 h after zymosan antiinflammatory M2-like macrophages (CD206^+^) and to a lesser degree eosinophils (Siglec F^+^) and dendritic cells (CD11c^+^, MHC II^+^) are found in the neighborhood of mast cells (**Figure 1D**). Quantitation of the presence of M2-like macrophages in the cellular neighborhood of mast cells was subsequently performed with independent MELC runs using a scoring system differentiating between random distribution (0) as well as more (1) or less (−1) often than expected for random distribution. Here, the presence of M2-like macrophages next to mast cells reached significance 48 h after zymosan injection (**Figure 1E**). Proinflammatory M1-like macrophages (CD86^+^) or neutrophils (Ly6G^+^) in the neighborhood of mast cells are found in numbers corresponding to a random distribution (**Figure 1D**).

**Figure 2:**
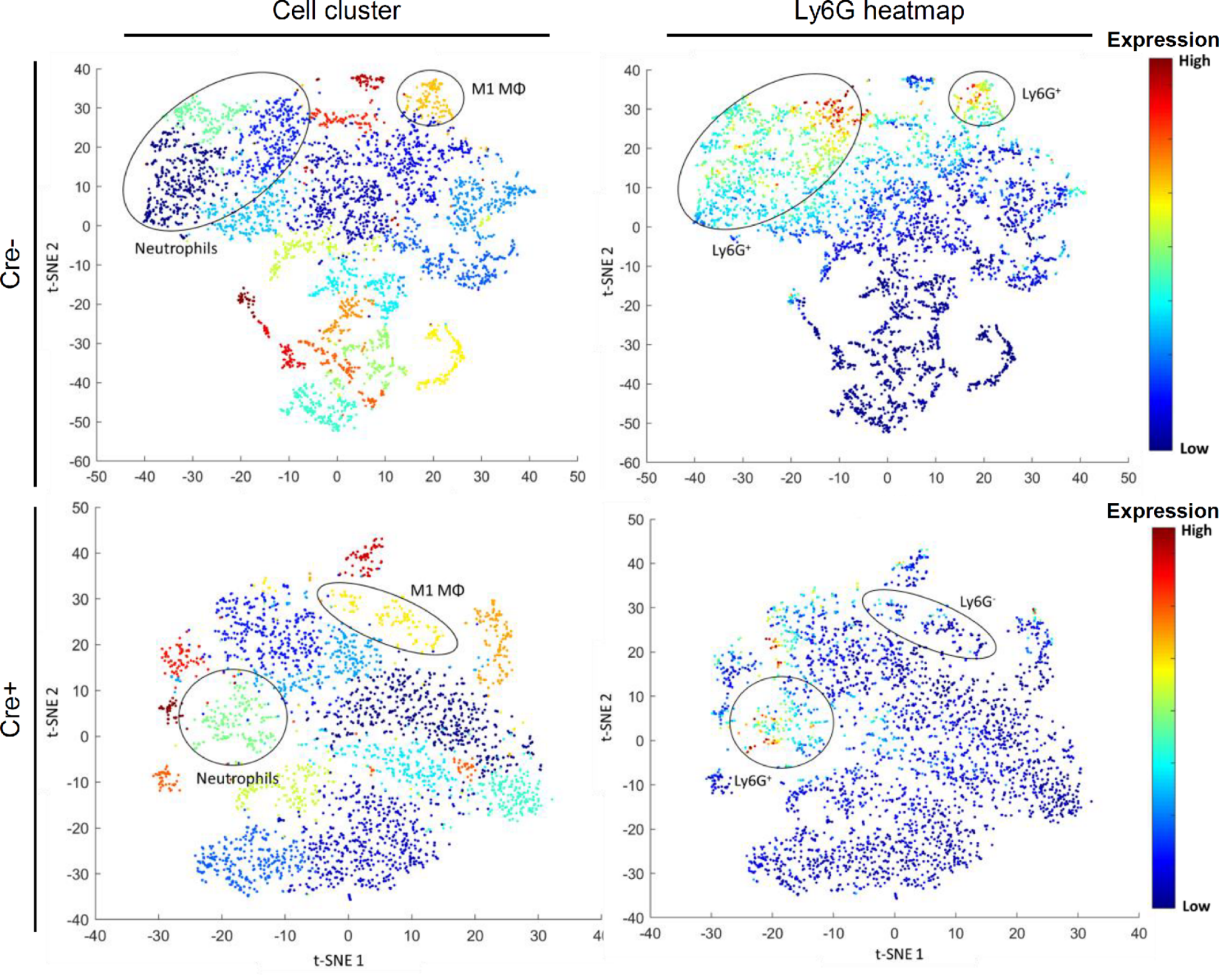
M1 macrophage phagocytosis of neutrophils is reduced in mast cell-deficient Mcpt5-DTA Cre^+^ mice compared to Cre^-^ control mice. Images show representative BH t-SNE analysis from Mcpt5-DTA Cre^+^ or Cre^-^ mice 48 h after injection of zymosan. Plots on the left are colored by cell clusters defined by PhenoGraph analysis (**Figure S4**). Plots on the right are heatmaps for the expression of the neutrophil marker Ly6G. The position of cell phenotype clusters containing CD86^+^ M1 macrophages (M1 MΦ) or neutrophils (Ly6G^+^/F4 80^-^) is indicated.

To assess potential effects of mast cells on the immune response, we compared in a first approach the expression patterns of macrophage populations using a PhenoGraph analysis of MELC images with inflamed paw tissue 48 h after zymosan-injection in Mcpt5-DTA-Cre mice. In these mice Cre recombinase is under control of the mouse mast cell protease 5 (Mcpt5) promotor and activates a Cre-dependent expression of the catalytically active diphtheria toxin A (DTA) subunit causing constitutive mast cell deficiency (15). Accordingly, remaining numbers of mast cells in paws from Cre^+^ mice were around 20% as compared to Cre^-^ mice (**Figure S2C**). The PhenoGraph analysis showed a striking reduction of the Ly6G marker for neutrophil granulocytes in M1-like macrophages of the mast cell-deficient mice (Cre^+^), which is suggestive for decreased efferocytosis (**Figure 2**, **Figure S4**). Notably, neutrophils were more present 4 h than 48 h after zymosan injection, while Ly6G-positive macrophages were more prominent 48 h after zymosan injection (**Figure S5**) reflecting increased efferocytosis during the resolution phase.

**Figure 3:**
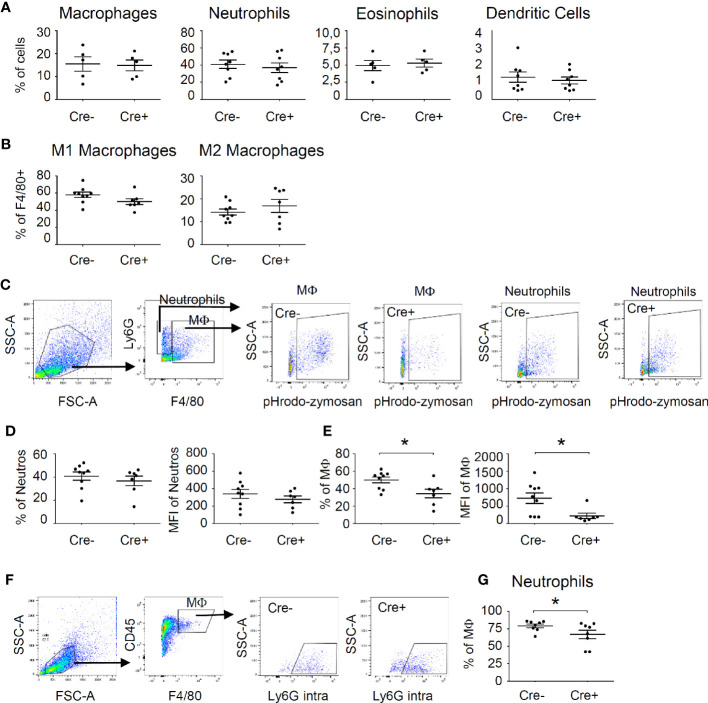
Mast cells influence phagocytosing activity of macrophages during resolution phase of zymosan-induced inflammation. **(A)** FACS analysis for the number of neutrophils, macrophages, eosinophils and dendritic cells in Mcpt5-DTA-Cre^-^ and Cre^+^ mice 48 h after injection of zymosan (10 µl, 12 mg/ml in PBS). Data are shown in % of all cells as mean ± S.E.M. (n=5–9), unpaired two-tailed t-test. **(B)** Number of M1-like (CD86^+^) macrophages and M2-like (CD206^+^) macrophages 48 h after injection of zymosan (10 µl, 12 mg/ml in PBS). Data are shown in % of all F4 80^+^ cells as mean ± S.E.M. (n=7–9), unpaired two-tailed t-test. **(C)** Gating strategy for flow cytometry analysis of phagocytosis of pHrodo Red zymosan by macrophages (F4 80^+^) and neutrophils (F4 80^-^/Ly6G^+^). **(D, E)** Phagocytosis of pHrodo Red zymosan (10 µl, 12 mg/ml in PBS) by neutrophils (panel D) or macrophages (panel E) in Mcpt5-DTA-Cre^-^ and Cre^+^ mice 48 h after zymosan injection. Data are mean ± S.E.M. (n=7–9), unpaired two-tailed t-test, *p<0.05. **(F)** Gating strategy for flow cytometry analysis of phagocytosis of neutrophils by macrophages. Intracellular staining of Ly6G^+^ neutrophils was performed. **(G)** Decreased phagocytosis of neutrophils (intracellular Ly6G) by macrophages (F4 80^+^). Data are mean ± S.E.M. (n=7–9), unpaired two-tailed t-test, *p<0.05.

The observed potential reduction of efferocytosis was not due to alterations in the recruitment of immune cells to the site of inflammation, since the number of neutrophils, macrophages, dendritic cells and eosinophils were not changed in mast cell-deficient mice 48 h after zymosan injection (**Figure 3A**, **Figure S6A**). Thus, mast cell-derived cytokines do not contribute to the recruitment of cells in that phase of the inflammatory reaction. Also, macrophage polarization toward M1-like or M2-like phenotypes was not altered by the absence of mast cells 48 h after zymosan injection (**Figure 3B**, **Figure S6B**). Finally, since mast cells are known to regulate the ability of macrophages to phagocytose apoptotic cells, such as neutrophils (30), we determined phagocytotic activity of macrophages and neutrophils by injecting pH-sensitive pHrodo Red zymosan bioparticles, which become fluorescent after phagocytosis inside of lysosomes. Forty-eight hours after injection of these pHrodo Red zymosan particles FACS analysis showed no difference in the percentage of for pHrodo Red zymosan^+^ neutrophils or the mean fluorescence intensity (MFI) in these cells between Cre^-^ and Cre^+^ Mcpt5-DTA mice (**Figures 3C, D**). Most importantly, the percentage and the MFI of pHrodo Red zymosan^+^/F4 80^+^ macrophages were significantly decreased in mast cell-deficient mice (**Figures 3C, E**). Likewise, the phagocytotic activity of macrophages toward neutrophils, as determined by the percentage of macrophages showing phagocytosis of neutrophils (Ly6G^+^), was decreased in mast cell-deficient mice (**Figures 3F, G**). Notably, the observed decreased phagocytotic activity of macrophages in mast cell-deficient mice did not lead to significant changes in the mechanical hypersensitivity between Cre^-^ and Cre^+^ Mcpt5-DTA mice (**Figure S7**), demonstrating that the decreased efferocytosis is not taking part in the nociceptive processes underlying mechanical hypersensitivity.

**Figure 4:**
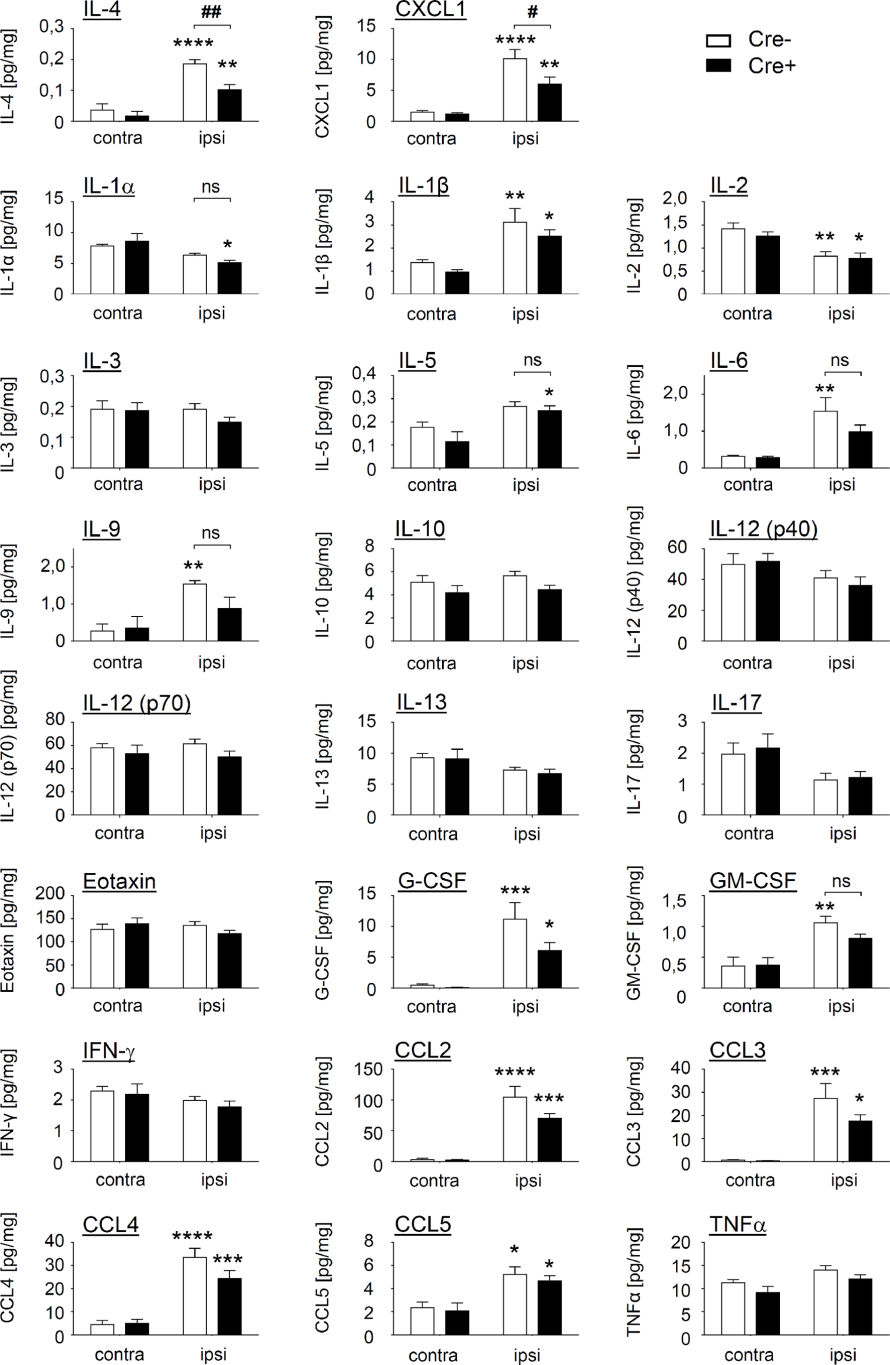
Mast cells influence the level of IL-4 and CXCL1 in paws of zymosan-injected mice. Levels of 23 cytokines, chemokines and growth factors in contralateral paws (untreated) and ipsilateral paws 48 h after injection of zymosan (10 µl, 12 mg/ml in PBS) in Mcpt5-DTA Cre^-^ and Cre^+^ mice determined by multiplex cytokine assay. Data are mean ± S.E.M. (n=6). Two-way ANOVA, Tukey’s multiple comparison test, significance between ipsi- and contralateral paws within one genotype is presented by *p<0.05, **p<0.01, ***p<0.001, ****p<0.0001, significance between genotypes is presented by ^#^<0.05, ^##^p<0.01.

To identify mediators, that mediate mast cell-regulated efferocytosis, we determined the levels of 23 cytokines, chemokines and growth factors in Cre^-^ and Cre^+^ Mcpt5-DTA mice. Out of 23 mediators included in the screen, 11 were significantly increased in the inflamed paws of Mcpt5-DTA-Cre- mice as compared to tissue from untreated mice (**Figure 4**). Out of these 11 mediators only IL-4 and CXCL1 showed a significant difference in zymosan-injected paws between Cre^-^ and Cre^+^ mice. Both mediators were significantly lower in zymosan-injected paws of mast cell-deficient mice. Other cytokines, such as IL-6 and IL-9, were only significantly elevated in zymosan treated paws from Cre^-^ mice, but did not reach significance between Cre^-^ and Cre^+^ mice (**Figure 4**). IL-4 is known for driving polarization of macrophages toward antiinflammatory (M2-like) phenotypes and CXCL1 for promoting chemotaxis of neutrophils. However, the decreased efferocytosis in mast cell-deficient mice cannot be explained by an increase of M1-like macrophages nor a decrease of neutrophil number, since neither of these effects was observed by flow cytometry analysis.

### RNA Sequencing Shows Activation of Type I IFN Pathway in Zymosan-Induced Mast Cells

**Figure 5:**
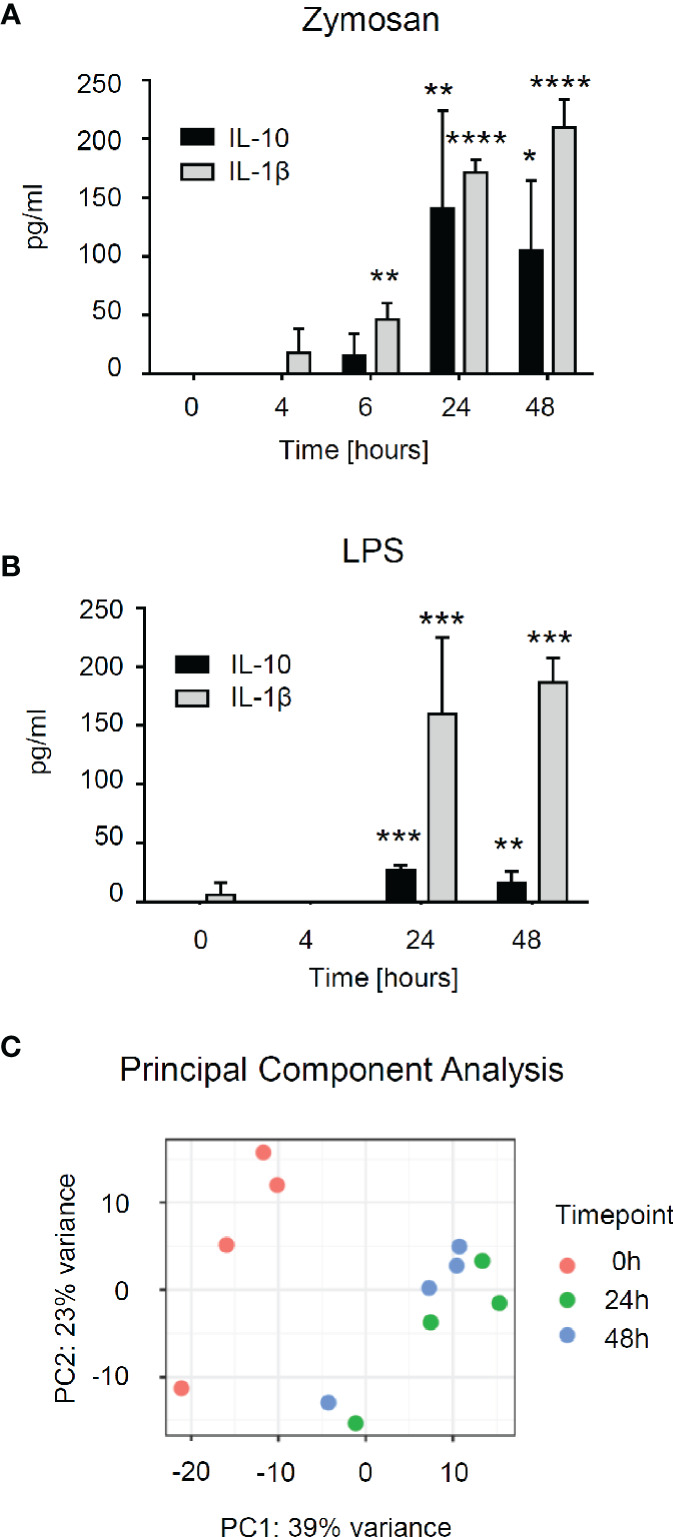
RNA sequencing shows differential expression of genes in bone marrow-derived mast cells (BMMCs) after induction with zymosan. **(A, B)** Concentration of IL-10 and IL-1β in the supernatant of BMMCs induced with zymosan (10 µg/ml) (panel A) or lipopolysaccharides (LPS) (100 ng/ml) (panel B) at the indicated time points. Data are mean ± S.E.M. (n=4). One-way ANOVA, Dunnett’s multiple comparison test for IL-10 and IL-1β, compared to 0 h control, *p<0.05, **p<0.01, ***p<0.001, ****p<0.0001. **(C)** The principal component analysis (PCA) was calculated on the 500 most variable genes across conditions in the RNA sequencing.

To better understand zymosan-induced changes in the gene expression of mast cells, we employed bone marrow-derived mast cells (BMMCs). First, we stimulated BMMCs with zymosan or LPS to determine the time course of their activation by determining the release of IL-10 and Il-1β. The levels of both cytokines reached a maximum after zymosan stimulation for 24–48 h (**Figure 5A**). A similar response was seen for LPS stimulation whereby the IL-10 release was markedly lower than during zymosan-stimulation (**Figure 5B**). Thus, to investigate the changes in gene expression induced by zymosan in mast cells in detail, we used untreated BMMCs as well as BMMCs stimulated for 24 or 48 h with zymosan, FACS-sorted them to homogeneity using antibodies against CD117 and FcεRIα (**Figure S8**) and performed the mRNA sequencing. The principal component analysis showed that the major differences in gene expression were seen between unstimulated and stimulated cells (**Figure 5C**). No significant differences in gene expression were detected between 24 and 48 h. Most of the significantly differentially expressed genes are upregulated at both time points when compared to unstimulated cells.

**Figure 6:**
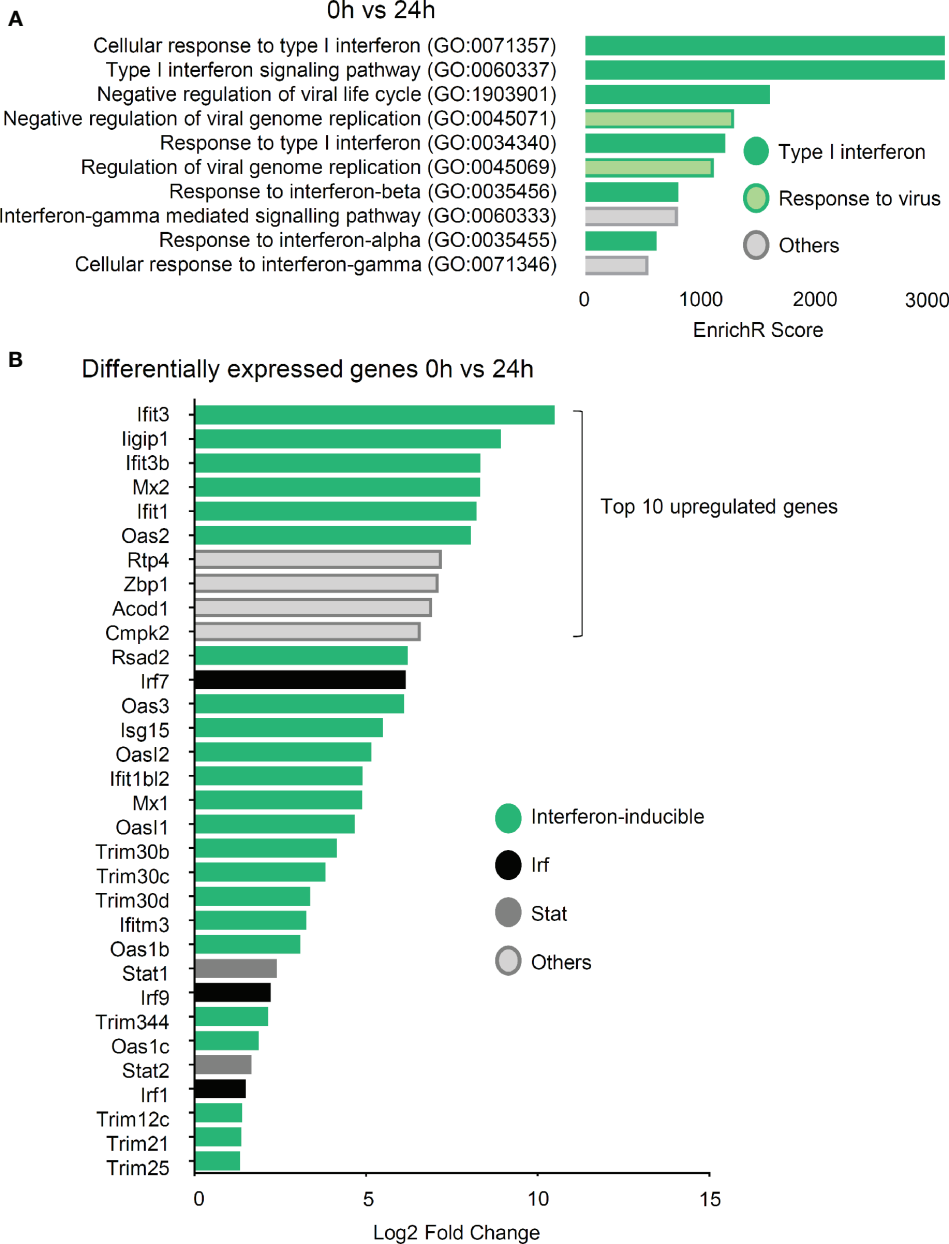
IFN type I signaling is the dominant response in bone marrow-derived mast cells (BMMCs) after 24 h stimulation with zymosan. **(A)** Top ten upregulated gene ontology terms of RNA sequencing by EnrichR 24 h vs. 0 h after induction of BMMCs with 10 µg/ml zymosan. **(B)** List of the 32 genes with the strongest upregulation 24 h after induction with 10 µg/ml zymosan as compared to untreated BMMCs.

Gene ontology term enrichment analysis of the sequencing data showed that the most prominent response is the upregulation of the type I IFN pathway after 24 and 48 h incubation (**Figure 6A**, **Figure S9**). Eight and nine groups out of the top ten gene ontology terms were related to this response after 24 and 48 h, respectively. (**Figure 6A**, **Figure S9**). The type I IFN response is generally regarded as an anti-viral response, which explains why terms related to “response to virus” are assigned to some genes. Moreover, after 24 h the six strongest upregulated genes were all IFN-inducible genes (**Figure 6B**). One of the genes with the strongest increase (6,12 log2 fold change) after 24 h of zymosan stimulation is Interferon regulatory factor 7 (Irf7), a transcription factor, which mediates the induction of the type I IFN response by TLR ligands in macrophages (12). Likewise, Irf1 and Irf9 are upregulated, with a log2 fold increase of 1.45 and 2.18, respectively. Thus, so far the data show that in mast cells zymosan induces the release of several antiinflammatory cytokines, and additionally a type I IFN response is triggered. Either of the responses alone or in combination can serve to explain the altered efferocytosis by macrophages in mast cell-deficient mice. mRNA expression for the two mediators, IL-4 and CXCL1, which were at the protein level significantly lower in inflamed paws from mast cell-deficient mice, was also detected in the zymosan-stimulated BMMCs, although changes between untreated and treated cells did not reach significance due to variability of the expression levels.

### Mast Cells Release of IFN-β in Response to Zymosan and LPS Requires Phagosome Maturation

**Figure 7:**
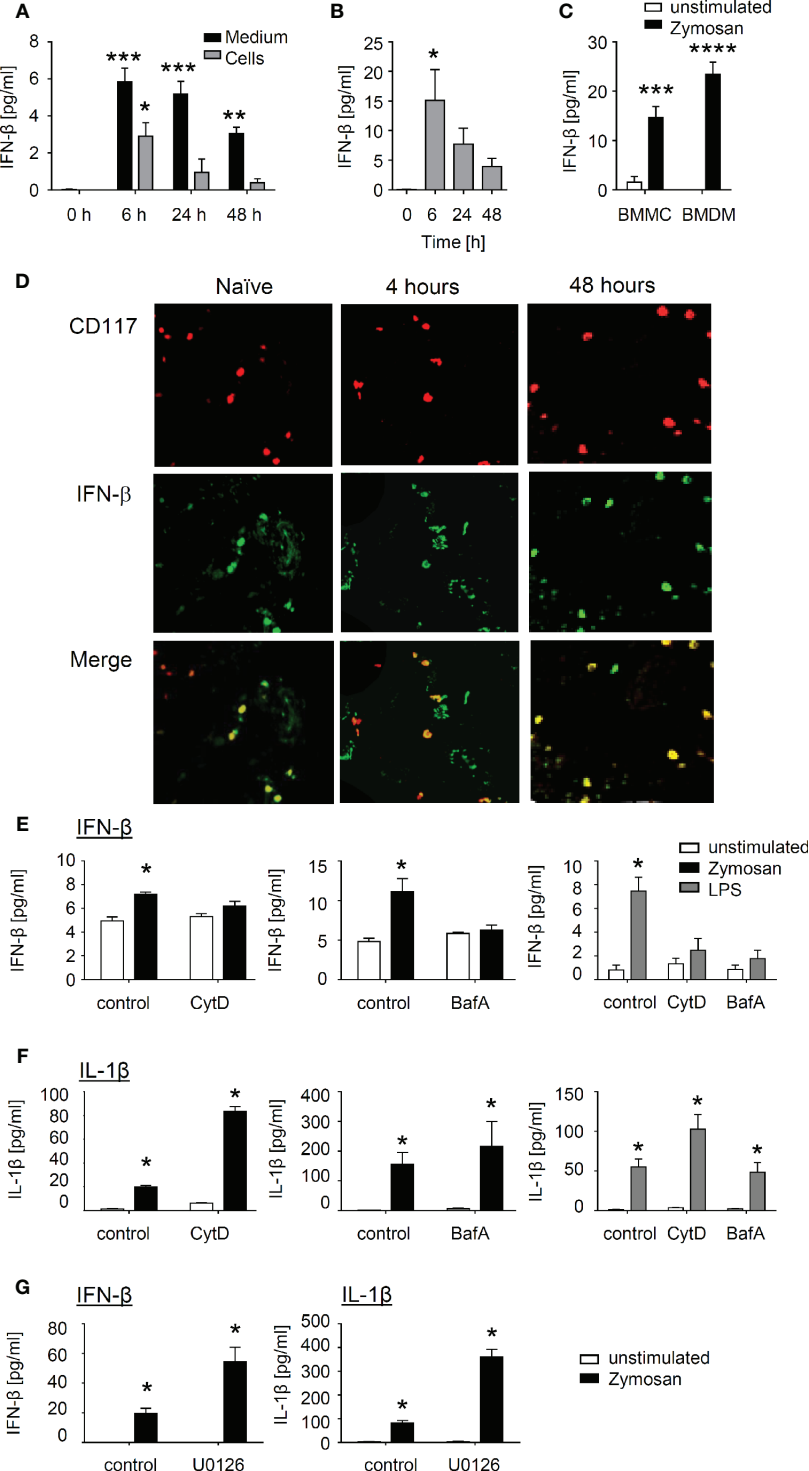
Interferon **(**IFN)-β is produced by mast cells after induction with zymosan or LPS and requires receptor internalization. **(A)** IFN-β from bone marrow-derived mast cells (BMMCs) after induction with zymosan (10 µg/ml) at the indicated time points in cell pellet and supernatant. Data are shown as mean ± S.E.M. (n=3). One-way ANOVA, Dunnett’s multiple comparison test compared to control, *p<0.05, **p<0.01, ***p<0.001. **(B)** Release of IFN-β from BMMCs after induction with lipopolysaccharides (LPS) (100 ng/ml) at the indicated time points. Data are shown as mean ± S.E.M. (n=3). One-way ANOVA, Dunnett’s multiple comparison test compared to control, *p<0.05. **(C)** Release of IFN-β from BMMCs and BMDMs 24 h after induction with zymosan (10 µg/ml). Data are shown as mean ± S.E.M. (n=4). Two-way ANOVA, Sidak’s multiple comparison test compared to unstimulated control, ***p<0.001, ****p<0.0001. **(D)** Representative stainings for CD117 and IFN-β of paws from naïve mice or 4 and 48 h after zymosan-injection. **(E, F)** BMMCs either untreated or pre-treated for 1 h with 25 µM Cytochalasin D or 50 µM Bafilomycin A were stimulated for 24 h with 10 µg/ml zymosan or 100 ng/ml LPS. The concentrations of IFN-β **(E)** or IL-1β **(F)** were determined in the medium. Data is shown as mean ± S.E.M. (n = 4). Multiple t-test comparing unstimulated and zymosan or LPS stimulated cells, Holm Sidak’s multiple comparison test, *p<0.05. **(G)** BMMCs either untreated or pre-treated for 1 h with 10 µM MEK-inhibitor U0126 were stimulated for 24 h with 10 µg/ml zymosan. The concentrations of IFN-β and IL-1β were determined in the medium. Data are shown as mean ± S.E.M. (n = 4). Multiple t-test comparing control and zymosan treatment, Holm Sidak’s multiple comparison test, *p<0.05.

Next, we aimed to validate the findings from the RNA sequencing approach by determining the concentration of type I IFNs released by BMMCs after induction with zymosan and LPS. The time course showed an early IFN-β increase in the medium (6 h after zymosan or LPS stimulation), which declined gradually afterwards (**Figures 7A, B**). In contrast, IFN-α, another member of the type I IFN-family, was not detected after stimulation of BMMCs (**Figure S10**). IFN-β concentrations in the mast cells themselves were not detectable in untreated BMMCs suggesting that IFN-β is newly synthesized (**Figure 7A**). It should be noted that the amount of IFN-β synthesized in BMMCs was in a similar range as the amount of IFN-β synthesized in bone marrow-derived macrophages, which served as positive control (**Figure 7C**). Importantly, immunohistological analysis showed that IFN-β is expressed in mast cells in paws from naïve mice as well as 4 and 48 h after zymosan injection into the paws of wild type mice (**Figure 7D**), suggesting that in contrast to BMMCs mast cells in paws show a basal IFN-β expression.

Since in macrophages TLR2- and TLR4-induced IFN-β release depends on the internalization of these receptors, we investigated if receptor internalization is also a prerequisite in mast cells (12, 13). First we tested whether or not IFN-β release depends in mast cells on the translocation of surface receptors to acidic endolysosomal compartments. We used Cytochalasin D (Cyt D) to prevent receptor internalization by inhibiting actin polymerization and Bafilomycin A (Baf A), a proton pump inhibitor, which interferes with endosomal acidification. Both inhibitors inhibited the release of IFN**-**β (**Figure 7E**), whereas the release of IL-1β in response to zymosan and LPS was not reduced (**Figure 7F**). Finally, zymosan has previously been demonstrated to activate various immune cells in an ERK-dependent matter through dectin-1 instead of TLR2 (31–33). However, inhibition of the ERK signaling pathway using the MEK inhibitor U0126 did not reduce zymosan-induced IFN-β release from BMMCs (**Figure 7G**).

## Discussion

Being located closely to host-environment interfaces, mast cells are among the first cells to come into contact with invading pathogens. They are equipped with a wide variety of receptors and an immense amount of preformed mediators and are thus able to respond quickly and specifically to diverse stimuli. Besides their role in initiating and promoting a proinflammatory immune response, they also have antiinflammatory and immunosuppressive functions. In this regard it has been proposed that mast cells are able to adjust their mode of action to the changing microenvironment during the different stages of an inflammation (34). Antiinflammatory immunomodulatory functions of mast cells have been so far mostly assigned to the cytokine IL-10 (35–38). It is well known that many cell types, including mast cells, respond to viral infections through TLR3, TLR7, and TLR9 initiating the type I IFN response as well as release of various proinflammatory cytokines (7, 8), which make them targets for antiinflammatory treatments including the use of cytokines of the IL-1 family with antiinflammatory properties (39, 40). RNA sequencing showed that mast cells are also able to initiate a strong type 1 IFN response in response to TLR2 and TLR4 activation. In this regard we observed a strong upregulation of genes associated with the type I IFN pathway in mast cells, stimulated with the TLR2 agonist zymosan as well as the release of IFN-β from these cells. This was surprising since mast cells were believed to be unable to internalize the receptor-pathogen complex necessary for IFN production and since TRIF signaling does not participate in LPS-mediated induction of TLR4 (13, 14). In contrast to TLR4, TLR2 is not associated with TRIF adaptor molecules and is known to be able to induce the type I IFN pathway in a MyD88-dependent way (10).

In our study we observed the release of IFN-β also after TLR4 induction by LPS, contradicting previous findings. A possible explanation for the induction of IFN-β by TLR4 despite the apparent absence of TRIF signaling might be that, similar to TLR2, TLR4 can mediate IFN-β induction in a MyD88-dependent way. The absence of detectable IFN-β in one previous study might be explained by the fact that IFN detection was not performed through a direct measurement, such as an ELISA assay, but through an indirect assay using IFN induced luciferase production in L929 cells transfected with an IFN-sensitive luciferase construct (14). Possibly this assay was not sensitive enough to detect IFN, since also viral activation of mast cells led not to IFN-β detection. In the study of Dietrich et al. (13), lack of TLR-induced IFN-β synthesis was proposed to be based on the inability of mast cells to actively internalize the pathogen-receptor complex. However, this is in contrast to several papers, which demonstrated the internalization of TLR4 receptor in mast cells. For example, LPS treatment of BMMCs induced the internalization of TLR4 receptor by a mechanism dependent on the activity of dynamin and the transport protein Huntingtin (41). Here, TLR4 internalization was necessary for LPS-induced ERK1/2 activation and TNFα production. Likewise, intracellular localization of TLR4 was shown for peripheral blood-derived mast cells, lung mast cells as well as in the human mast cell line HMC-1 (42, 43).

Previous studies demonstrated that endolysosomal trafficking is required for the successful induction of the type I IFN pathway for primarily cell surface-located TLRs. It was also shown that TRAF3 is necessary for induction of the type I IFN response and is not efficiently recruited to the TLR signaling complex at the plasma membrane. The intracellular localization together with potential conformational changes in the acidic environment are thought to facilitate the interaction with TRAF3 (11, 44, 45). In accordance with this, pharmacological blockage of receptor internalization and endosome maturation in mast cells also inhibits the induction of type I IFNs, while the proinflammatory response is still active upon TLR2 and TLR4 activation.

The time course of the release of IFN-β from BMMCs showed that the IFN-β release peaks 6 h after zymosan stimulation, while significant amounts of IL-10 and IL-1β are only detected after 24 h of zymosan treatment. Since IFN-β was not detected in unstimulated mast cells, this early response demonstrates the induction of IFN-β synthesis. The induction of the expression of other mediators, which are able to induce IFN-β, would be also a possibility, although this scenario does not fit to the observed time frame. Taken together the data point toward a direct induction of IFN-β synthesis in BMMCs by zymosan. However, it should be noted that the situation *in vivo* differs from the findings in BMMCs, since we found mast cells expressing IFN-β not only in inflamed paws but also in naïve mice. The fact that mast cells in paws express IFN-β also under basal conditions points toward the storage of this mediator for its fast release in response to pathogens including zymosan or LPS. Thus, the specific *in vivo* microenvironment of mast cells in paw tissue apparently allows the basal expression and most likely the storage of IFN-β in mast cells. While IFN-β was seen in mast cells *in vivo* using immunohistochemistry, we did not detect IFN-β by ELISA in whole paw lysates. However, since relative few mast cells are localized in the paw, IFN-β levels might be locally increased but too low to be detected in the context of whole tissue analyses. Whether or not this IFN-β released by mast cells affects phagocytotic activity of macrophages during zymosan-induced inflammation is not clear. Also in case that IFN-β modulates the phagocytotic activity of macrophages in this model, it can only be speculated if this regulation is a direct effect or is mediated by secondary mechanisms, such as altering the release of other mediators from mast cells themselves or other immune cells is still an open question, which needs to be addressed in future studies.

The enhancing effect of IFN-β on efferocytosis by macrophages has been shown recently, demonstrating the pro-resolving functions of this cytokine (46). Here, we observed a decrease of the phagocytotic activity of macrophages in mast cell-deficient mice during resolution of inflammation, while no changes in the number of neutrophils, monocyte-derived macrophages, eosinophils or dendritic cells recruited to the zymosan-injected paw were observed. These findings show that mast cells do not play a dominant role in immune cell recruitment in the resolution phase of zymosan-induced inflammation. The screening for alterations in cytokine and chemokine levels in mast cell-deficient mice showed a decrease of IL-4 and CXCL1 in absence of mast cells. CXCL1 is best known for its ability to promote chemotaxis of neutrophils. The fact that CXCL1 levels are reduced in mast cell-deficient mice without that a change in neutrophil numbers occurs supports the notion that mast cells are not involved in immune cell recruitment in this phase of inflammation. Instead other CXCL1 functions, such as its role in wound healing (i.e. epithelialization and angiogenesis) (47), might explain its mast cell-dependent increase during the resolution phase of inflammation. IL-4 on the other hand is known to drive polarization of macrophages toward antiinflammatory (M2-like) phenotypes and fits to the localization of mast cells in the cellular neighborhood of M2-like macrophages. Other cytokines, which are released by mast cells, were not changed 48 h after zymosan injection in Cre^+^ mast cell deficient mice as compared to Cre^-^ mice. In this regard, although IL-10 and PGE_2_ synthesis in BMMCs was induced by zymosan there was no difference between their levels in inflamed paws from mast cell-deficient and control mice (**Figure 4**, **Figure S11**). On one hand this could reflect the differences between the phenotypes of BMMCs and mast cells located in the paw tissue. On the other hand IL-10 can also be produced by other immune cells in amounts, which could mask changes in mast cell-derived IL10 in the context of whole tissue analyses. Notably, this notion does not oppose possible local effects of mast cell-derived IL-10 in the defined area surrounding the zymosan-containing area. Another explanation could be based on IFN-β effects on mast cells themselves, which could influence the synthesis and release of mediators. In this regard also other mediators that can be produced by mast cells upon zymosan-stimulation, i.e. IL-1β, IL-4 and PGE_2_, have been demonstrated to increase phagocytosis in macrophages as well (48–50). Therefore, the enhancing effect of mast cells on the phagocytosing activity of macrophages could be derived from one of these mediators, including IFN-β, or a combination of these (51).

Finally, the observed localization of mast cells during the resolution phase of local inflammation within an antiinflammatory cellular neighborhood suggests a reversal of the immunological function of mast cells. In this regard various mediators including cytokines (e.g. IL-10 and IL-4), chemokines (e.g. CXCL1) or lipids such as PGE_2_, which all can mediate effects promoting antiinflammation and/or wound healing, have already been demonstrated to be synthesized in mast cells and to promote mast cell-dependent antiinflammatory effects (36, 52). Thus, the well-known proinflammatory role of mast cells in the beginning of an inflammation seems to be reversible allowing mast cells to gain an antiinflammatory phenotype and to expand its role in inflammation depending on the needs of the specific microenvironment.

## Data Availability Statement

The datasets presented in this study can be found in online repositories. The data are available in the GEO database with the accession number GSE165523.

## Ethics Statement

The animal study was reviewed and approved by Regierungspräsidium Darmstadt. Written informed consent was obtained from the owners for the participation of their animals in this study.

## Author Contributions

LK, SP, and JC did the *in vivo* experiments and performed FACS analyses. LK and TSchi performed mRNA sequencing. SE and IE processed and analyzed the mRNA sequencing data. NF and DT provided LC-MS/MS analysis data. AW, BB performed FACS sorting. TSchä and AK performed MELC analyses. KS and LK designed experiments and wrote the paper. All authors contributed to the article and approved the submitted version.

## Funding

This work was supported by the DFG grants SCHO817/3-3, SFB1039 (TPA08, B04, B06 and Z01) and GRK2336 (TP01, TP06, TP07). The Else Kröner-Fresenius-Foundation (EKFS) funded JC as part of the Else-Kröner Graduateschool. This research was supported by the research funding program Landes-Offensive zur Entwicklung Wissenschaftlich-ökonomischer Exzellenz (LOEWE) of the State of Hessen, Research Center for Translational Medicine and Pharmacology TMP, and Research Center for Translational Biodiversity Genomics (TBG).

## Conflict of Interest

SE was employed by Institute of Molecular Biology GmbH.

The remaining authors declare that the research was conducted in the absence of any commercial or financial relationships that could be construed as a potential conflict of interest.
