# Supplementary material for: Bacterial and Fungal Toll-Like Receptor Activation Elicits Type I IFN Responses in Mast Cells
Source: Front Immunol. 2021 Feb 12;11:607048. doi: 10.3389/fimmu.2020.607048 (PMC7907501; doi:10.3389/fimmu.2020.607048)
Supplement: Supplementary file 1 [file DataSheet_1.pdf]

Supplementary Figure S1:

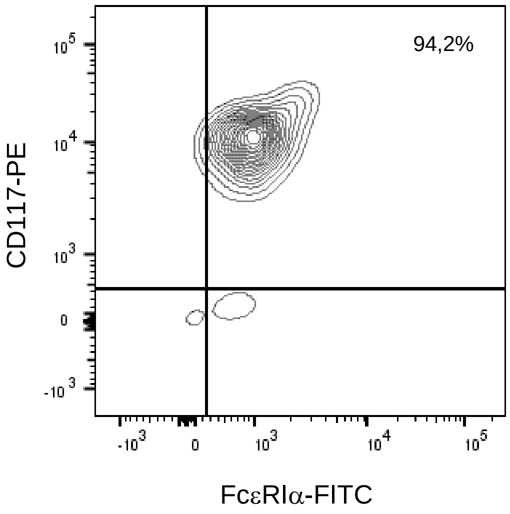

Figure S1. Purity of BMMCs grown according to standard protocols was assessed by staining for CD117+ FcεRIα+ cells and flow cytometry analysis.

Supplementary Figure S2:

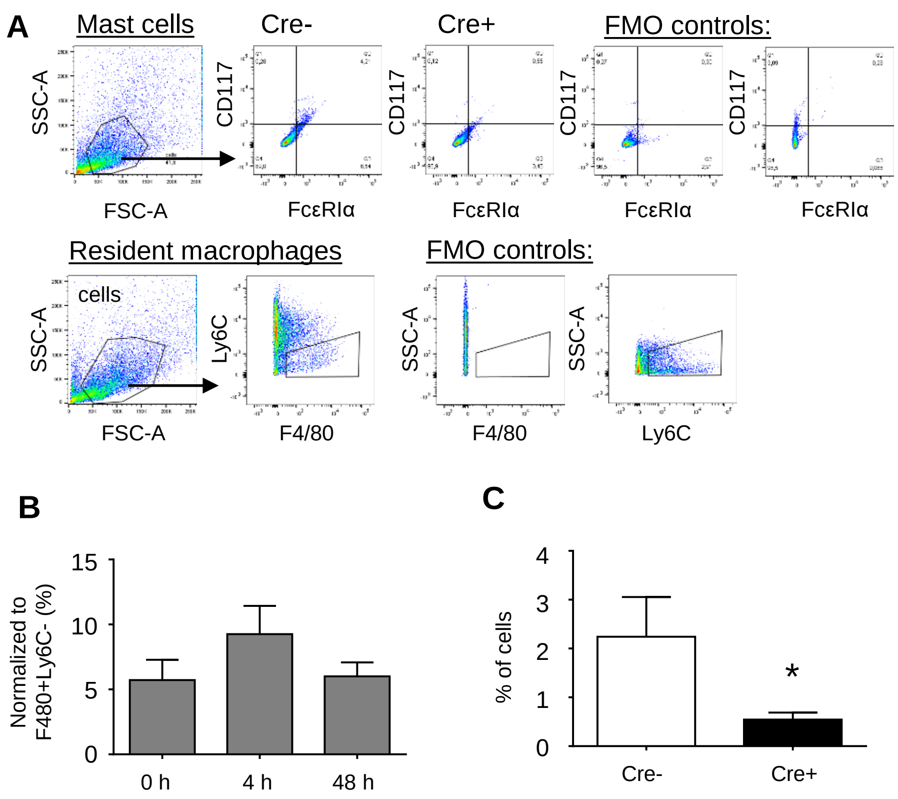

Figure S2. Mast cells numbers are decreased in paws of Mcpt5-DTA Cre$^+$ mice as compared to Cre$^-$ mice. **(A)** Mast cells were identified as CD117$^+$/FcεRIα$^+$ cells by flow cytometry, resident macrophages were used for normalization and identified as F4/80$^+$/Ly6C$^-$ cells. FMO controls for CD117, FcεRIα, F4/80 and Ly6C are shown. **(B)** Flow cyometry analysis of mast cells in paws of Mcpt5-DTA Cre$^-$ after injection of 10 µl zymosan (12 mg/ml) at indicated time points (n=4). Number of mast cells were normalized to resident macrophages (F4/80$^+$/Ly6C$^-$). Data are shown as mean ± S.E.M., one-way ANOVA, Dunnett's multiple comparison test. **(C)** Flow cytometry analysis of mast cells in paws of Mcpt5-DTA Cre$^-$ and Cre$^+$ mice (n=4). Data is shown as mean ± S.E.M., unpaired t-test, one-tailed, *p<0.05.

Supplementary Figure S3:

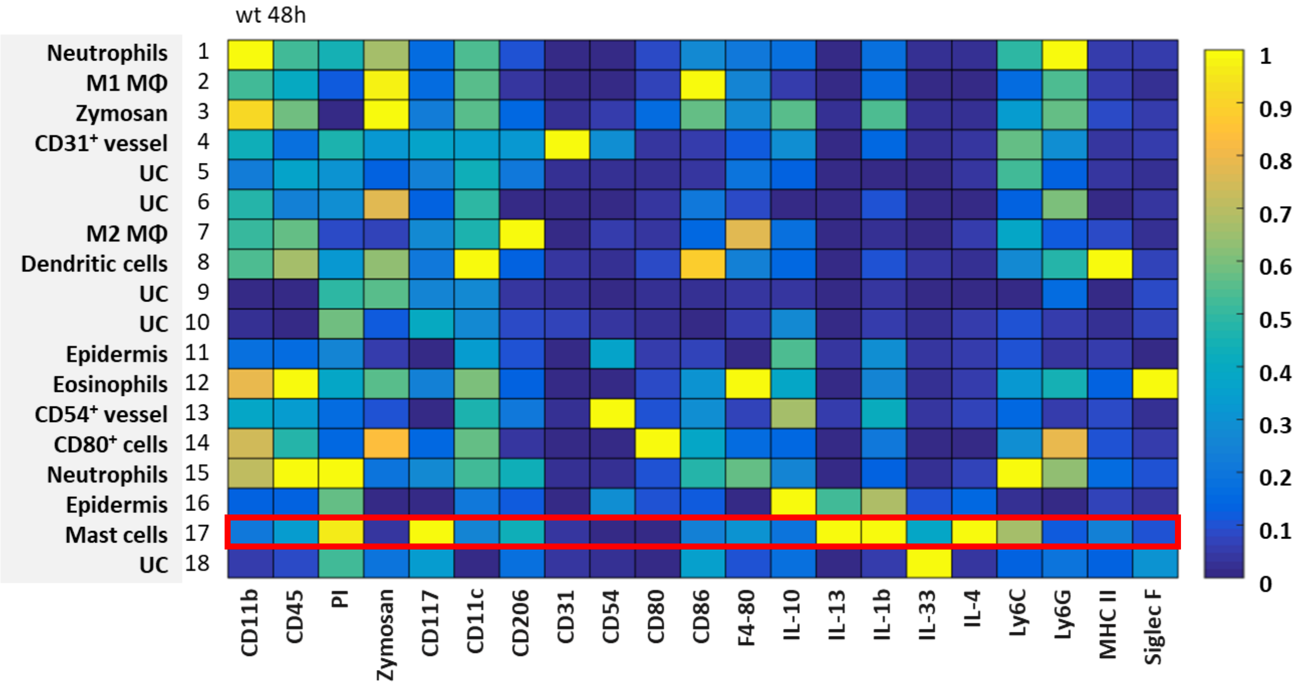

Figure S3. Representative PhenoGraph analysis of MELC images from mouse paw 48 h after zymosan-injection shows expression of antiinflammatory cytokines (IL-13, IL-33, IL-4) and of proinflammatory IL-1β in mast cells. The heat map shows a PhenoGraph analysis of MELC data (n=4) based on single-cell segmentation with CD45 and nuclei marker using CellProfiler. Yellow depicts strong expression and dark blue low expression, relative within each column. Marker expression in mast cells is marked by a red box. MΦ, macrophages; UC, unidentified cluster.

Supplementary Figure S4:

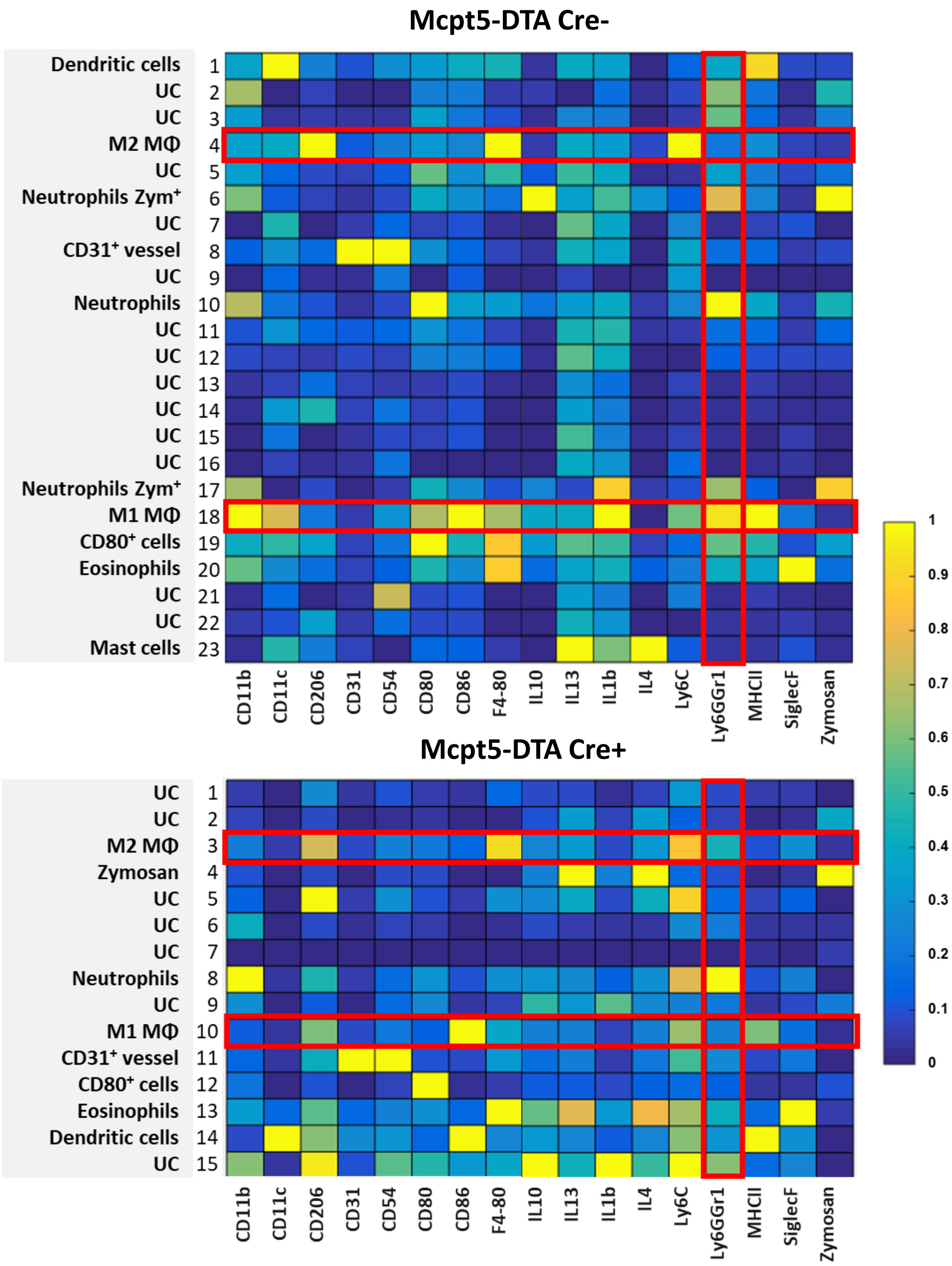

Figure S4. Representative PhenoGraph analysis of MELC images from Mcpt5-DTA Cre^{+} and Cre^{-} mouse paw 48 h after zymosan-injection. Heat maps show PhenoGraph analysis of MELC data (n=3, respectively) based on single-cell segmentation with CD45 and nuclei marker using CellProfiler. Yellow depicts strong expression and dark blue low expression, all relative within one column. Marker expression in macrophage phenotype cluster (M1, M2) is marked by a horizontal red box. Expression of neutrophil marker Ly6G is marked by a vertical red box. MΦ, macrophages; UC, unidentified cluster.

Supplementary Figure S5:

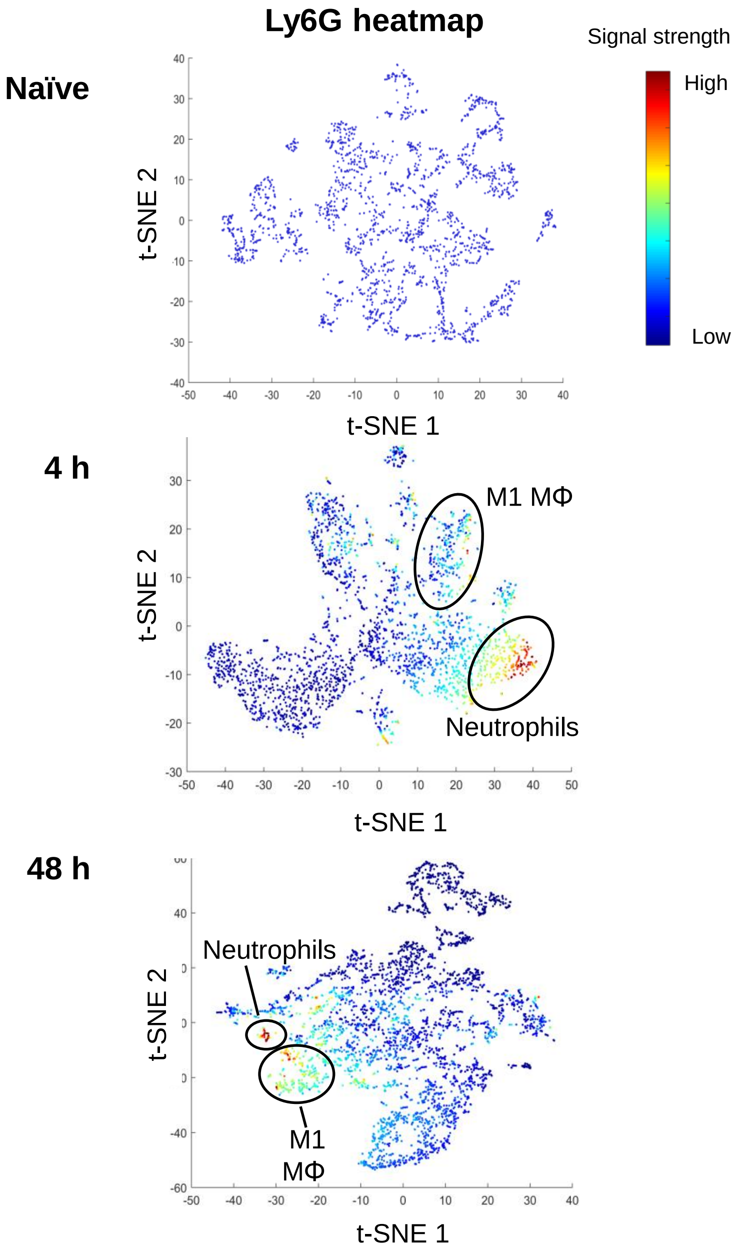

Figure S5. Representative BH t-SNE analysis of MELC images from mouse paw naïve, 4 h or 48 h after zymosan-injection. Shown is the time course of neutrophil occurrence based on Ly6G marker expression (n=3, respectively). Neutrophil and M1 macrophage phenotype cluster are indicated by cycles in t-SNE plots. Ly6G-positive macrophages at 4 h are less prominent as compared to 48 h reflecting the increased efferocytosis during resolution of inflammation. Single-cell segmentation is based on CD45 and nuclei marker using CellProfiler. MΦ, macrophages.

Supplementary Figure S6:

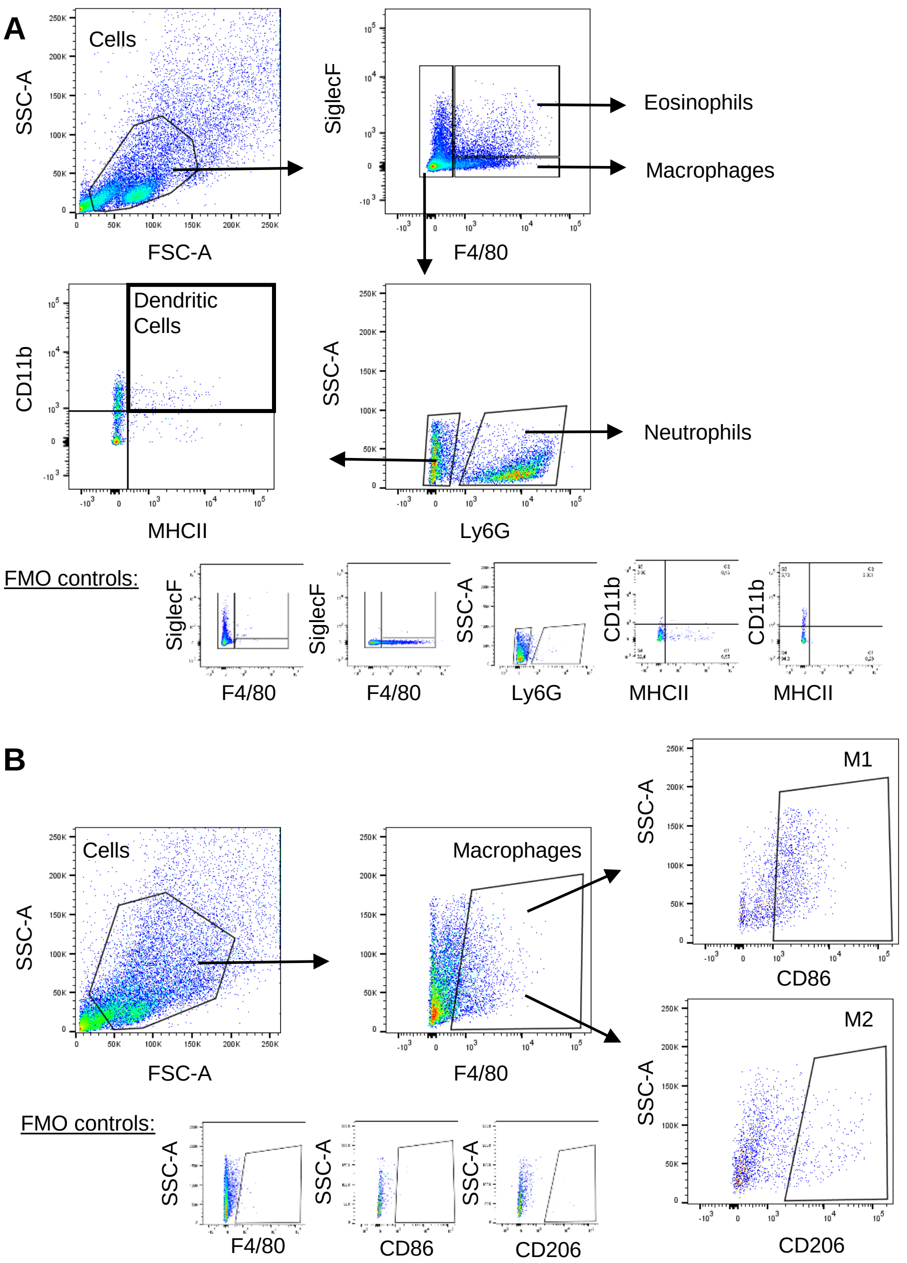

Figure S6. Gating strategy for immune cells and their subtypes in paws of Mcpt5-DTA Cre- and Cre+ mice 48 h after injection of zymosan (10 µl, 12 mg/ml). **(A)** Cells were gated based on their side scatter area (SSC-A) and forward scatter area (FSC-A). Within, F4/80+ and F4/80- populations were identified. Within the F4/80+ cells, eosinophils were identified as SiglecF+ and macrophages were identified as SiglecF-. Within the F4/80- cells, neutrophils were identified as Ly6G+, while Ly6G- cells were further analyzed for CD11b+ MHCII+ dendritic cells. FMO controls are shown below the gating strategy. **(B)** M1 macrophages were identified as CD86+ cells among F4/80+ macrophages and M2 macrophages were identified as CD206+ cells among F4/80+ macrophages. FMO controls are shown below the gating strategy.

Supplementary Figure S7:

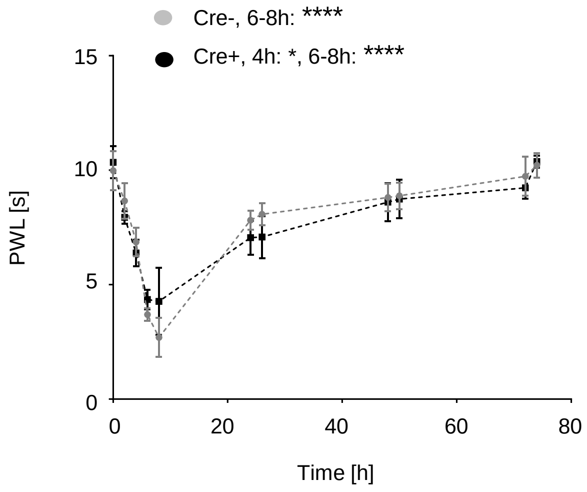

Figure S7 Paw withdrawal latencies of Mcpt5-DTA Cre^- and Cre^+ mice during zymosan-induced inflammation. Mechanical paw withdrawal latencies in Mcpt5-DTA Cre- and Cre+ mice (n=5) at indicated time points after injection of zymosan (10 µl, 12 mg/ml). Data are shown as mean ± S.E.M., two-way ANOVA, Tukey's multiple comparison test, *p<0.05, ****p<0.0001 compared to 0 h.

Supplementary Figure S8:

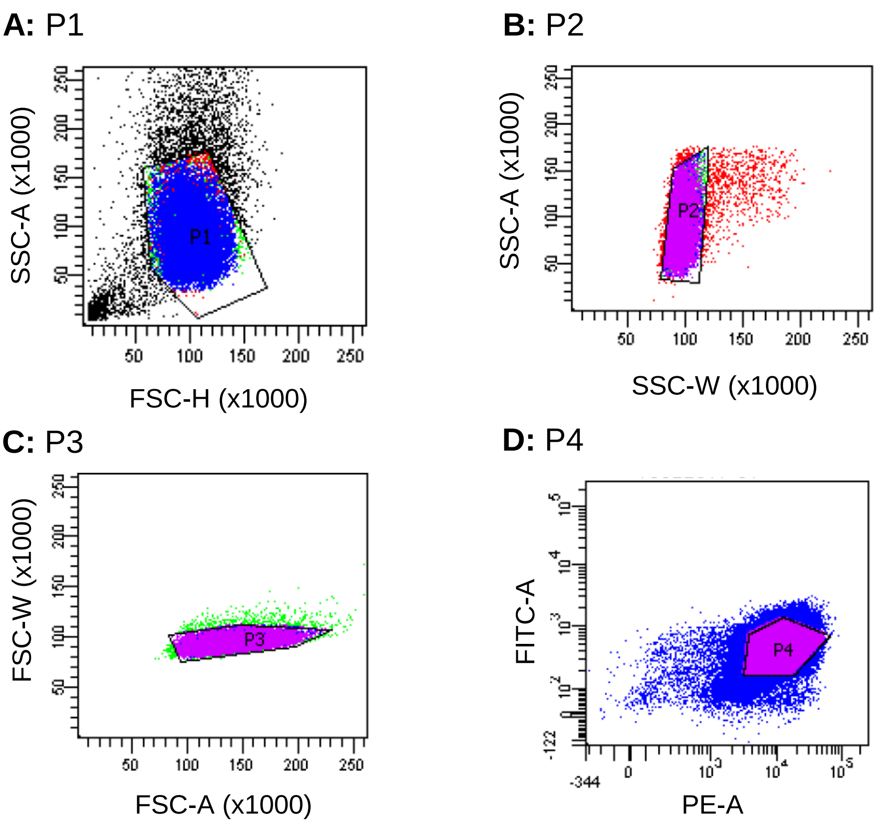

E

Tube: 01

Population	#Events	%Parent	%Total
All Events	100,000	####	100.0
P1	79,829	79.8	79.8
P2	74,194	92.9	74.2
P3	70,551	95.1	70.6
P4	55,047	78.0	55.0

Figure S8. Gating strategy for FACS sorting of mature BMMCs for RNA sequencing. **(A)** Cells were gated based on their side scatter area (SSC-A) and forward scatter height (FSC-H) in P1. **(B)** Single cells were then identified within P1 based on their side scatter area (SSC-A) and side scatter width (SSC-W) in P2, followed by **(C)** their forward scatter width (FSC-W) and forward scatter area (FSC-A) in P3. **(D)** Mature mast cells were identified within P3 as CD117+ FcεRIα+ cells. **(E)** Exemplary data of percentage of mature mast cells (P4) sorted for subsequent RNA sequencing.

Supplementary Figure S9:

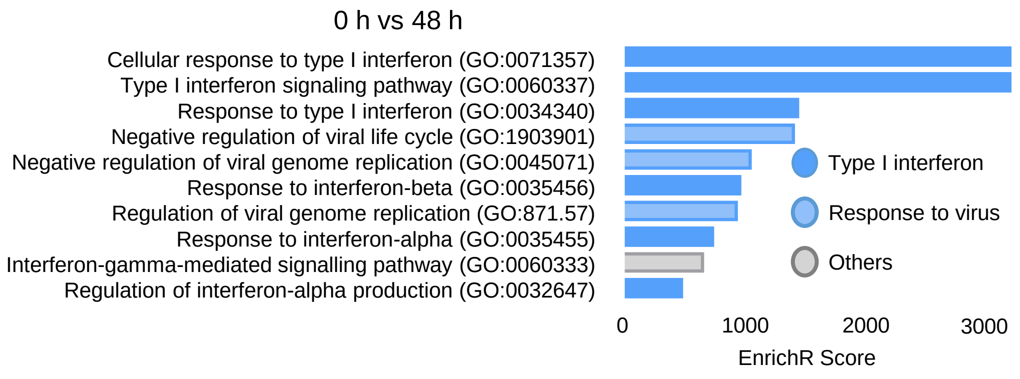

Figure S9. Top-ten upregulated gene ontology terms of RNA sequencing by EnrichR 0 h vs 48 h after induction of BMMCs with 10 µg/ml zymosan.

Supplementary Figure S10:

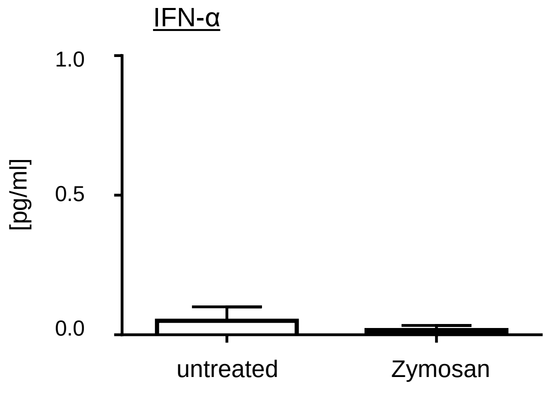

Figure S10. IFN-α is not detectable in supernatant of BMMCs 24 h after induction with 10 µg/ml zymosan. Data is shown as mean ± SEM.

Supplementary Figure S11:

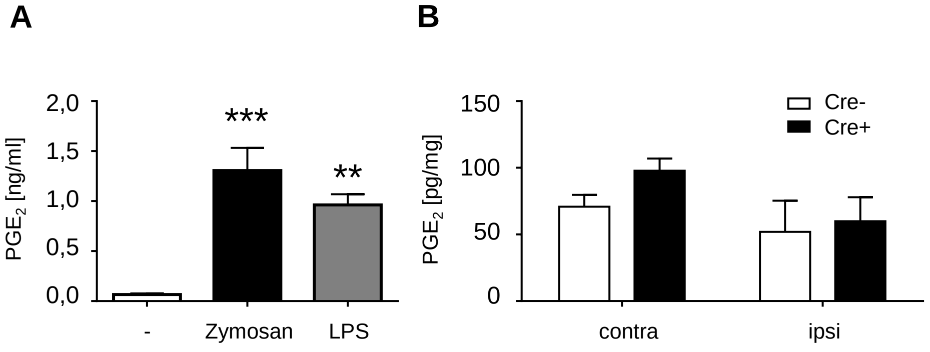

Figure S11. PGE_2 release by mast cells. **(A)** Concentration of PGE_2 (n=5) after 24 h of induction with zymosan (10 µg/ml) or LPS (100 ng/ml). Data are shown as mean ± S.E.M. One-way ANOVA, Dunnett's multiple comparison test compared to control, $^{**}p<0.01$, $^{***}p<0.001$. **(B)** Figure1: PGE_2 level in contralateral and ipsilateral paws 48 hours after zymosan injection. Data are shown as mean ± S.E.M. (n=5).
